# The Value(s) of Vaccination: Building the Scientific Evidence According to a Value-Based Healthcare Approach

**DOI:** 10.3389/fpubh.2022.786662

**Published:** 2022-03-09

**Authors:** Giovanna Elisa Calabro', Elettra Carini, Alessia Tognetto, Irene Giacchetta, Ester Bonanno, Marco Mariani, Walter Ricciardi, Chiara de Waure

**Affiliations:** ^1^Section of Hygiene, University Department of Life Sciences and Public Health; Catholic University of the Sacred Heart, Rome, Italy; ^2^VIHTALI (Value In Health Technology and Academy for Leadership & Innovation), Spin-Off of Università Cattolica del Sacro Cuore, Rome, Italy; ^3^Department of Prevention, Azienda ULSS 6 Euganea, Padova, Italy; ^4^Department of Medicine and Surgery, University of Perugia, Perugia, Italy

**Keywords:** value, vaccination, vaccines, value-based healthcare, allocative value, technical value, personal value, societal value

## Abstract

**Objectives:**

To provide a new value-based immunization approach collating the available scientific evidence on the topic.

**Methods:**

Four value pillars (personal, allocative, technical, and societal) applied to vaccination field were investigated. A systematic literature review was performed querying three database from December 24th, 2010 to May 27th, 2020. It included studies on vaccine-preventable diseases (VPDs) that mentioned the term value in any part and which were conducted in advanced economies. An in-depth analysis was performed on studies addressing value as key element.

**Results:**

Overall, 107 studies were considered. Approximately half of the studies addressed value as a key element but in most of cases (83.3%) only a single pillar was assessed. Furthermore, the majority of papers addressed the technical value by looking only at classical methods for economic assessment of vaccinations whereas very few dealt with societal and allocative pillars.

**Conclusions:**

Estimating the vaccinations value is very complex, even though their usefulness is certain. The assessment of the whole value of vaccines and vaccinations is still limited to some domains and should encompass the wider impact on economic growth and societies.

## Introduction

Optimization of the use of limited available resources has been one of the main topics of discussion in healthcare so far. Over the past few years, different techniques have been developed to solve the problem of healthcare systems sustainability including evidence-based decision making (to ensure that only interventions with strong evidence of effectiveness and cost-effectiveness are used), quality improvement, and cost reduction (1). The value-based healthcare approach also emerged in order to address this issue with the aim to increase the value that is derived from the resources available for the population (2).

Michael Porter, in 2010, introduced the concept of value in healthcare describing it as the “health outcome achieved per dollar spent” (3). Given the characteristics of European health systems that aim to ensure equity, universality and solidarity to all, efficacy, and efficiency parameters highlighted by Porter's value paradigm are necessary but not sufficient (4). Allocation of resources should guarantee the highest level of quality and value in respect to the principles of equity in finance and distribution, hence a paradigm shift in the analysis of value was proposed by Sir Muir Gray (5), with the aim of integrating the programming of health services to the approaches of modern population medicine (5). The suggested triple value paradigm identified allocative, technical, and personal values as cornerstone of healthcare delivery (6). Subsequently, in 2019, the Expert Panel on Effective Ways of Investing in Health (EXPH) of the European Commission proposed a comprehensive concept built on four value-pillars to define “value(s)-based healthcare” for conveying the guiding principles of solidarity-based healthcare systems (1):

– The personal value in terms of appropriate care to achieve patients' personal goals;
– The allocative value, namely the equitable distribution of resources across all patient groups;
– The technical value meant as the achievement of the best possible outcomes with available resources;
– The societal value that is represented by the contribution of healthcare to social participation and connectedness.

Among health technologies, vaccines are one of the most successful of contemporary times. In the last 50 years, worldwide, the elimination and the control of many diseases was made possible by vaccines which drastically reduced mortality rate and related complications. According to the World Health Organization (WHO), vaccinations prevent 2–3 million deaths every year (7). Vaccinations also contribute to the sustainability of health systems thanks to the reduction in hospitalizations and required medical treatment, visits, and examinations and, therefore, direct medical costs (8). Moreover, through the avoidance of the disease occurrence and its consequences, vaccination promotes the overall economic growth of countries and counters poverty (9, 10). Despite these important benefits vaccinations are currently challenged by several concerns, such as unequal access, lack of resources, and vaccine hesitancy. An exploitation of the broad value of vaccines and vaccination could help contrasting such concerns and strengthening vaccination policies and strategies.

The aim of this study is to identify and systematically describe how the value of vaccine(s) and vaccination has been addressed in the academic literature considering the four EXPH value pillar framework. The description of the scientific evidence on the whole value of vaccination will be useful to foster a new value-based immunization approach.

## Methods

A systematic review of the academic literature was carried out bearing in mind the four pillars of value (personal, allocative, technical, societal) as defined by the document by the EXPH of the European Commission (1). The relevant dimensions considered within each pillar were based on a comprehensive reading of EXPH report (1) and were as follows:

– Personal value: clinical and patients reported outcomes and experience measures, citizens' involvement and empowerment;
– Allocative value: access to vaccination, equity in provision, affordability, unwarranted variations, innovation;
– Technical value: health technology assessment (HTA) and models for assessing the economic evaluation of vaccines/vaccinations;
– Societal value: impact on population's wellbeing, productivity, and social cohesion; indirect and community protection; accountability of the decision-making process.

The systematic review was conducted and reported according to the Preferred Reporting Items for Systematic Reviews (PRISMA) (11).

### Search Strategy

For each pillar, a systematic review was carried out. Hence, four systematic reviews were conducted. PubMed and Web of Science databases were queried. For the technical value pillar, the University of York's Center for Reviews and Dissemination (CRD) database was also used.

The search strings—reported as Supplementary Materials—were launched on May 27th 2020. The systematic review was performed from December 24th, 2010 onwards as Michael Porter' article “What is value in health care?” was published December 23, 2010 (3). As described in introduction, this article was the first scientific contribution on the value of vaccination according to the conceptual framework of EXPH.

The articles records were entered in a work sheet and screened according to the inclusion/exclusion criteria.

### Inclusion/Exclusion Criteria

Studies were considered eligible if they dealt with vaccine-preventable diseases (VPDs), were conducted in advanced economies according to the list of the International Monetary Fund^1^ and mentioned the term “value” at least once in the entire text. The choice of including only studies conducted in advanced economies was made assuming that the basic necessities are fulfilled and thus the meaning of value could be more comparable. Only studies written in English were included. Commentary, editorials, conference presentation, and references not provided with full text were excluded as well as studies conducted in animals or *in vitro*.

### Selection Process and Data Extraction and Synthesis

Five researchers (E.C., A.T., I.G., E.B., M.M.) independently screened titles and abstracts first and full texts afterwards. Any disagreement was resolved by discussion or by the involvement of two other researchers (G.E.C., C.d.W.).

The following data were extracted from all the articles eventually included: author, year, country, type of study, study objective (single vaccine/vaccination strategy), study aim, target population, setting, vaccination strategy (mandatory/recommended/other), vaccination policy (free/co-payment/out of pocket), centrality of the value in the paper (main object of the study or not). Papers addressing the value as main object were further analyzed in order to collect information about the domains considered within each pillar. The additional information collected to disclose the different dimensions were as follows:

– Personal value: clinical outcomes (efficacy, effectiveness, safety etc.), patients' reported outcomes (PROMs, PREMs, satisfaction, quality of life), patients' reported experience measures (preference, attitudes, perceptions, awareness, knowledge, confidence, convenience, complacency, wellbeing, productivity, etc.), citizens' involvement, and empowerment in vaccination (participation, engagement, empowerment, commitment);
– Allocative value: accessibility (people possibility to obtain the services when needed)^2^, equity (absence of unfair and avoidable or remediable differences in vaccination provision among population groups)^3^, affordability (payment for health−care services must be based on the principle of equity, ensuring affordable services for all), appropriateness (i.e., effectiveness of the service for a particular type of patient, from which the patient could find a benefit), unwarrantedvariations (overuse, underuse), innovation (in terms of organizational program or strategy setup to improve vaccination coverages);
– Technical value: economic model (cost of illness, cost effectiveness, cost benefit, cost utility, budget impact, fiscal impact, other), general drivers of costs (direct healthcare costs, direct non−healthcare costs, indirect costs, intangible costs, tax revenue), vaccination/vaccine−related drivers of costs (vaccine safety, vaccine efficacy, costs of vaccine administration, costs of vaccination program), and HTA;
– Societal value: population's wellbeing (productivity, social cohesion, etc.), indirect/community protection (herd immunity, social responsibility, etc.), shared decision-making process on (accountability, transparency, appraisal criteria, monitoring of the impact of the decision appraisal, etc.), and Health Impact Assessment (HIA).

Data were included in Tables 1–4 and summarized descriptively through frequencies.

**Table 1:** Personal value as main object of the study (present in objectives/methods).

**First author, year, country**	**Study objective**	Vaccine^*^	**Clinical outcome**	**Patients reported outcome**	**Patients reported experience measures**	**Citizen's involvement and empowerment in vaccination**
Attwell K, 2015. Australia (12)	Vaccination strategy				Attitudes, perceptions	
Baumann A, 2019. Denmark (13)	Vaccine	HPV			Attitudes, perception, knowledge	
Böhm R, 2015. Germany (14)	Vaccination strategy				Attitudes, behavior	
Brown DS, 2014. USA (15)	Vaccine	HPV			Behavior, awareness, attitudes, willingness to pay	
Cataldi JR, 2019. USA (16)	Vaccination strategy				Universalism, benevolence, conformity, tradition, safety, self-direction	
Dunn AG, 2015. USA, Australia (17)	Vaccine	HPV			Perception	Participation, empowerment
Gidengil C, 2012. USA (18)	Vaccination strategy				Preference, attitudes, convenience	
Lavail KH, 2012. USA (19)	Vaccine				Confidence, attitudes, perceptions, preference, awareness	
Luyten, 2019. UK (20)	Vaccine				Perception, awareness	
Marshall, 2016. Australia (21)	Vaccine	MenB			Attitude, preference	
Nowak GJ, 2017 (22)	Vaccine	Influenza			Perception, awareness, knowledge, preference	Empowerment
Schoeppe J, 2017. USA (23)	Vaccination strategy				Knowledge, awareness, attitudes	Participation, engagement, empowerment
Steens, 2020. Norway (24)	Vaccination strategy				Importance, safety, effectiveness, confidence, conflicts with basic values	
Timmis, Rigat, 2017 (25)	Vaccine		Mortality, morbidity			
van der Putten, 2016 (26)	Vaccine		Mortality, morbidity	Quality of life		

* *This information has been reported only for papers addressing a specific vaccine/vaccination; HPV, human papilloma virus; MenB, meningococcus B*.

**Table 2:** Allocative value as main object of the study (present in objectives/methods).

**First author, year, country**	**Study objective**	Vaccine^*^	**Accessibility**	**Equity**	**Affordability**	**Appropriateness**	**Unwarranted variations**	**Innovation**
Attwell K, 2015. Australia (12)	Vaccination strategy		No	No	No	No	No	“I Immunize” campaign used community advocates and appealed to local values around social justice, parenting, and alternative lifestyles
Audisio RA, 2016. Europe (27)	Vaccination strategy	HPV	Yes	Yes	Yes	Yes	No	Extension of vaccination to boys
Bell S, 2020. UK (28)	Vaccination strategy	Influenza	No	No	No	No	No	Opt-out policy which asked healthcare workers to declare the reasons for declining
Bloom DE, 2015 (29)	Vaccine		No	No	Yes	No	No	No
Connolly T, 2012. USA, Singapore (30)	Vaccination strategy		No	No	No	No	No	Use of Internet to improve decision making around vaccination choices
van der Putten, 2016 (26)	Vaccine		No	Yes	Yes	No	No	No

* *This information has been reported only for papers addressing a specific vaccine/vaccination; HPV, human papilloma virus*.

**Table 3:** Technical value as main object of the study (present in objectives/methods).

**First author, year, country**	**Study objective**	Vaccine^*^	**Type of study/economic model**	**Drivers of costs**	**Innovative vaccination/vaccine-related costs drivers**
Audisio RA, 2016. Europe (27)	Vaccination strategy	HPV	Review on cost-effectiveness data		
Bacci JL, 2019. USA (31)	Vaccination strategy		Value-based incentive model for immunizations		Incentive earnings for pharmacies
Barbieri M, 2016. Belgium, France, UK, Germany, Italy, Spain, Sweden, the Netherlands (32)	Vaccination strategy		Review on cost-effectiveness data		
Bärnighausen T, 2012. USA, South Africa (33)	Vaccine	HPV	Cost-benefit	Direct healthcare costs, indirect costs, intangible costs	Behavior-related productivity gains (vaccination improves health and survival, and changes individual behavior, i.e., lowering fertility or increasing investment in education); externalities (improved outcomes in unvaccinated community members, e.g., through herd effects and reduction in the development of resistance to antibiotics)
Bloom DE, 2015 (29)	Vaccine		Review on challenges of economic evaluations		
Boccalini S, 2013. Italy (34)	Vaccine	Pneumococcal	Cost-effectiveness	Direct healthcare costs, intangible costs	
Brogan, 2017. USA (35)	Vaccination strategy	Influenza	Cost-effectiveness	Direct healthcare costs, indirect costs	Herd protection
Cafiero-Fonseca ET, 2017 (36)	Vaccine	Pneumococcal	Review on the full benefit of vaccination		
Damm O, 2015. Germany (37)	Vaccine	Varicella, HZV	Review on cost-comparison, cost-effectiveness, and cost-utility		
de Waure C, 2012 (38)	Vaccine	Influenza	Review on cost-effectiveness and cost-benefit		
Dort T, 2018. Belgium (39)	Vaccine	Rotavirus	Quality of care–isocurve model, budget optimization model	Direct healthcare costs	
Favato G, 2012. Italy (40)	Vaccination strategy	HPV	Cost of illness and cost-effectiveness	Direct healthcare costs	
Favato G, 2013. Italy (41)	Vaccination strategy	HPV	Real option model for cost-effectiveness	Direct healthcare costs	
Herlihy N, 2016 (42)	Vaccine	HPV	Economic surplus produced by vaccination	Direct healthcare costs	Costs of vaccine developing, manufacturing, marketing, procurement, vaccine purchasing, and delivering
Hoshi S, 2018. Japan (43)	Vaccination strategy	aP-containing vaccine	Cost-effectiveness	Direct healthcare costs	
Hoshi S, 2013. Japan (44)	Vaccination strategy	Pneumococcal	Cost-effectiveness	Direct healthcare costs, indirect costs	Costs of the vaccination program
Jiang Y, 2015. France (45)	Vaccination strategy	Pneumococcal	Public health and budget impact	Direct healthcare costs	
Jiménez E, 2015. Norway (46)	Vaccination strategy	HPV	Cost-effectiveness	Direct healthcare costs, indirect costs	Herd immunity
Kotirum S, 2017 (47)	Vaccine	Rotavirus	Review on cost-effectiveness, cost-benefit, and cost-utility analysis		
Lee BY, 2011. USA (48)	Vaccination strategy	Influenza	Cost-benefit from the patient perspective	Direct healthcare costs, indirect costs	Time for vaccination
Lee BY, 2015. USA (49)	Vaccination strategy	Influenza	Cost of illness	Direct healthcare costs, indirect costs	
Lee BY, 2012. USA (50)	Vaccination strategy	Influenza	Cost of illness	Direct healthcare costs, indirect costs	
Lee BY, 2012. USA (51)	Vaccine	Influenza	Cost- effectiveness and budget impact	Direct healthcare costs, indirect costs	
Lee BY, Shah M, 2012. USA (52)	Vaccine	Influenza	Review on economic value		
Leidner AJ, 2019. USA, Canada (53)	Vaccine		Review on economic evaluation and cost-effectiveness		
Ozawa S, 2016. USA (54)	Vaccine		Cost of illness and full income model	Direct healthcare costs, indirect costs	
Park M, 2018. UK (55)	Vaccine	HPV	Cost benefit	Direct healthcare costs, indirect costs	
Philipson TJ, 2017. USA (56)	Vaccine		Economic model for addressing the social value (total surplus)	Direct healthcare costs	Vaccine manufacturers' profit margin
Postma MJ, 2015. Germany, UK, France (57)	Vaccine		Position paper on full economic and societal value		
Preaud E, 2014. EU-27 (58)	Vaccination strategy	Influenza	Model for estimating public health and economic impacts	Direct healthcare costs, indirect costs	
Reyes JF, 2016. Australia (59)	Vaccination strategy	Rotavirus	Cost-utility	Direct healthcare costs	
Rheingans, 2014 (60)	Vaccine	Diarrheal vaccines	Review on economic evaluation		
Timmis, Rigat, 2017 (25)	Vaccine		Survey and focus study to identify a set of core value to evaluate vaccines		
Tully SP, 2011. Canada (61)	Vaccination strategy	HPV	Cost-utility	Direct healthcare costs	Vaccine cross-protection
van der Putten, 2016 (26)	Vaccine		Four step process based on review and a survey to identify and prioritize the broader economic impact of vaccines		
Waye, 2015. Canada (62)	Vaccine	Pneumococcal	Cost of illness	Direct healthcare costs	Indirect protection

* *This information has been reported only for papers addressing a specific vaccine/vaccination; HPV, human papilloma virus; HZV, herpes zoster virus; aP, acellular pertussis*.

**Table 4:** Societal value as main object of the study (present in objectives/methods).

**First author, year, country**	**Study objective**	Vaccine^*^	**Population's wellbeing**	**Indirect/community protection**	**Shared decision-making process**
Asgary A, 2012. Canada (63)	Vaccine	Influenza	Willingness to pay bids for a pandemic vaccine		
Barnighausen T, 2014. USA, South Africa (10)	Vaccine		Outcome-related productivity gains; behavior-related productivity gains; economic externalities	Community health externalities; herd immunity	
Bloom DE, 2015 (29)	Vaccine		Clinical outcome; avoided costs of care and lost income; higher productivity and earnings; lower rates of absenteeism	Herd effects, i.e., reduced usage of antibiotics and slower development of antibiotic resistance	
Cafiero-Fonseca ET, 2017 (36)	Vaccine	Pneumococcal	Averted direct medical costs and of informal care; improved health; enhanced labor market output	Herd effects or slowed pace of antimicrobial resistance	
Connolly T, 2012. USA, Singapore (30)	Vaccination strategy				Internet-based, interactive decision support
Postma MJ, 2015. Germany, UK, France (57)	Vaccine		Productivity	Herd immunity, social responsibility	
Timmis, Black, 2017 (64)	Vaccine				Transparency, accountability
van der Putten, 2016 (26)	Vaccine		Reduction in school days lost, increased productivity, and participation	Impact on other diseases, community externalities on diseases outbreak investigation and prevention, and households' welfare	
Schoeppe J, 2017. USA (23)	Vaccination strategy			Social change	Increased knowledge of local and state vaccination rates, familiarity with vaccine hesitancy, and understanding the concept of herd immunity

* *This information has been reported only for papers addressing a specific vaccine/vaccination*.

## Results

The overall research in the three databases yielded a total of 9,714 articles across the four pillars. After duplicates removal, 7,122 articles were screened based on title and abstract. In total, 704 articles were assessed for eligibility and 107 articles were eventually included. Of these, 78 (72.90%) were primary researches, 16 (14.95%) reviews, and 13 (12.15%) systematic reviews. Sixty (56.1%) articles tackled more than one pillar of the value. In particular, 44 (44.1%) addressed two pillars, 13 (12.1%) three pillars, and 3 (2.8%) all four pillars.

Sixty studies (56.1%) analyzed one or more vaccines, while 47 (43.9%) analyzed a vaccination strategy. For those studies about vaccines, 15 focused on influenza (25.0%), nine on pneumococcal vaccines (either conjugate or polysaccharide) (15.0%), seven on Human papilloma virus (HPV) (11.7%), four on multiple vaccines (6.67%), three on Herpes zoster virus (HZV) (5.0%), three on rotavirus (5.0%), one on Meningococcus B (MenB), one on mumps, and one on Norovirus (1.7% each); for 16 studies the vaccine was not specified (26.7%). As for the 47 studies focused on a vaccination strategy, eight regarded influenza (17.0%), eight pneumococcal vaccines (either conjugate or polysaccharide) (17.0%), eight HPV (17.0%), six multiple vaccines (12.8%), three HZV (6.4%), one pertussis (2.1%), one rubella (2.1%), and one rotavirus (2.1%); 11 studies did not specify the vaccination strategy (23.4%). Most of the studies were conducted in the USA (*n* = 27, 25.27%); six (5.61%) in Japan; five (4.67%) in the UK; five (4.67%) in Australia and five (4.67%) in Italy; four (3.74%) in Germany and Canada each; three (2.80%) in Spain, three in Belgium (2.80%) and three (2.80%) in Norway; 20 (18.69%) studies had multiple countries as a setting, while for 17 (15.89%) the country setting was not specified; the remaining five (0.93%) studies were conducted in Denmark, France, Korea, New Zealand, Sweden.

Value was the main objective of the research in 54 studies (50.5%), nine among them addressing more than one value pillar. A description of the main findings for each pillar is reported hereafter.

### Personal Pillar

Fifteen studies addressed personal value as key element (Table 1). Of these, eight were primary researches (75.9%), four reviews (13.8%), and three systematic reviews (10.3%).

Eight studies (53.3%) did not mention any specific vaccine (12, 14, 16, 19, 20, 23, 25, 26), three focused on HPV (20%) (13, 15, 17), two addressed childhood vaccination in general (13.3%) (18, 24), one MenB vaccination (21), and one seasonal influenza (6.7% each) (22).

Only two studies out of 15 (13.3%) took into consideration clinical outcomes (25, 26) in terms of impact on mortality and morbidity, whereas most of the articles (86.7%) reported information about patients' reported experience measures (12–24). Eventually, the issue of citizens' involvement and empowerment was assessed in three out of 15 articles (20.0%) (17, 22, 23). Patients' experience was analyzed mainly in terms of knowledge (13, 22, 23), perceptions/beliefs (12, 13, 16–22, 24), attitudes (12–14, 16, 18, 19, 23), and preferences (15, 18, 19, 21, 22). Citizens' involvement and empowerment in vaccination was investigated in terms of empowerment (22, 23) and engagement (12, 23).

### Allocative Pillar

The search brought a total number of six articles addressing allocative value as main objective, among which three (50%) were primary studies (12, 28, 30) and three (50%) reviews (26, 27, 29). The studies were very heterogeneous in terms of vaccines taken into account and only few dealt with a specific vaccination, namely influenza in one case (16.7%) (28), and HPV in another one (16.7%) (27).

None of the included articles addressed the issue of unwarranted variations. Three (50%) papers addressed the topic of the affordability (26, 27, 29), whereas four (66.7%) articles brought the attention on innovative aspects in terms of organizational delivery (28), informational model (12, 30) or extension of the vaccination to previously excluded populations (27). Equity was addressed by two (33.33%) studies (26, 27), while only one (16.67%) tackled accessibility and appropriateness (27).

### Technical Pillar

Thirty-six studies addressed the technical value as a key element. The majority (eight studies; 22.22%) of the studies concerned influenza vaccines (35, 38, 48–52, 58) and HPV (eight studies; 22.22%) (27, 33, 40–42, 46, 55, 61), five pneumococcal vaccines (13.9%) (34, 36, 44, 45, 62), three rotavirus (8.3%) (39, 47, 59), while six (16.7%) multiple vaccines (31, 32, 37, 43, 56, 60). Eventually, six (16.67%) did not address any specific vaccines in particular (25, 26, 29, 53, 54, 57).

With respect to specific dimensions considered within the pillar, a total of 19 (52.8%) papers focused on the cost-effectiveness, including also the cost-utility (25, 27, 32, 34, 35, 37, 38, 40, 43, 44, 46–48, 50, 53, 56, 59–61). Six studies (16.7%) addressed the cost of illness (48–50, 54, 58, 62), two (5.6%) the cost-benefit (33, 55), and one (2.8%) the budget impact (45). The study by Jiménez et al. (46) was a HTA report addressing also current use of the technology, technical characteristics, and the clinical effectiveness. Four papers (11.1%) considered alternative ways/models to assess the economic value of vaccination (31, 39, 41, 42). One article assessed the economic surplus produced by vaccination defined as the sum of all health and economic benefits of vaccination, minus the costs of vaccine development, production, and distribution (42). One study developed a novel approach (using the payoff method) to value real options in health care (41). Quality of Care (QoC)-Isocurve Model and budget optimization model was used by one study (39), while one developed a new value-based incentive payment model for immunizations delivered in community pharmacies (31). Eventually, four studies (11.1%) either described or tried to identify the broader potential economic impacts of vaccination (26, 29, 36, 57). In particular, Postma et al. (57) stressed the difficulty of assessing the full economic (and societal) value of the vaccination and suggested that wider economic and societal values should be integrated or at least considered in the economic values of vaccination programs. Lifelong benefits for individuals, the reduction of indirect costs, and improved quality of life should not be underestimated and should be captured as an integral part of the economic assessment of vaccination programs (57). Bloom et al. suggested an urgent need of concerted efforts to identify datasets that could estimate the full benefits of vaccination programs and fit the challenges. High-quality data are needed to support research to “enjoy gains in public health that are more transformational than incremental” (29).

### Societal Pillar

Nine studies analyzed value as main objective in the societal pillar. Five (55.6%) were primary studies (10, 23, 30, 57, 63), three (33.3%) reviews (26, 29, 64), and one (11.1%) was a systematic review (36). Study vaccinations were influenza in one paper (11.1%) (63) pneumococcal in another one (11.1%) (36) and HPV (11.1%) (10). Eventually, in six studies (66.7%) the study vaccination was not reported (23, 26, 29, 30, 57, 64).

Concerning the specific dimensions considered within the pillar, the topics addressed were population's wellbeing (seven studies, 77.8%) (10, 23, 26, 29, 36, 57), indirect/community protection (five studies, 55.5%) (10, 26, 29, 36, 57, 63), and shared decision-making (three studies, 33.3%) (23, 30, 64). Health Impact Assessment was not performed by any of the selected studies.

Population's wellbeing was indirectly described as gain of productivity (10, 26, 29, 57), healthy aging (57), as well as through social cohesion in terms of awareness of the population of being part of a community and of contributing to it (23, 36, 57). Of note, Barnighausen spoke about “outcome-related productivity,” which considers direct health care costs or loss of productive time and “long term mental, physical, or cognitive impairments” and “behavior-related productivity gains” brought by vaccination (10). Also benefits from reduction in the development of drug resistance was mentioned (10, 36). Finally, shared decision-making was proposed by three articles in different forms: a set of core evaluation criteria to be applied to the vaccine evaluation procedure, with the ultimate aim to improve the accountability in vaccine decision-making (64); a community intervention to reduce vaccine hesitancy by providing pro-vaccine parents with tools to “engage in positive dialogue about immunization in their community,” so that pro-vaccine parents were empowered to be immunization advocates (23); and with the use of an interactive, internet-based decision tool that could help parents taking informed decisions about vaccination for their children (30).

## Discussion

Although the real and tangible benefits of vaccines are recognized globally, in many countries a decline in coverages has been observed in recent years and is still ongoing. Concerns or fears about vaccines safety, lack of trust, social norms, myths undermining confidence in vaccines, failure by some healthcare to provide evidence-informed advice, barriers in the access to vaccines (e.g., poor availability, co-payments), and failure to understand the underlying mechanisms that decrease vaccination confidence are some of the barriers to vaccination (1). The understanding of the broad value of vaccination and the effective translation of this knowledge to the different stakeholders is therefore important to strengthen vaccination policies and strategies and counteracting disinformation and, misinformation.

This systematic review has shown that the value of vaccine(s) and vaccination(s) was addressed by a few number of papers in the last 10 years. Only 54 out of the initial 7,122 articles (0.8%) addressed the topic in depth. The most studied vaccines were seasonal influenza, pneumococcal, and HPV vaccines. Most of the papers included in the systematic review addressed the technical value and then the personal one, while the allocative and societal values were addressed in very few papers. These results are noteworthy as it calls the attention on the need for more research on these dimensions that are relevant for fully addressing the value of vaccination also from the societal perspectives.

Nonetheless, it should be pointed out that the assessment of the allocative and societal pillars poses several challenges. In fact, the allocative value encompasses many dimensions that cannot be assessed in a standardized way and are context-specific. The role played by equity, accessibility and appropriateness within the immunization agenda 2030 (65) and also in respect to COVID-19 vaccines emphasizes the importance of their evaluation (66).

As far as the societal value is concerned, several interesting suggestions for the development and the application of new methods and criteria emerged in respect to the impact of vaccination on social cohesion and Health Impact Assessment (AMR). In particular, with respect to AMR, vaccinations are still undervalued, even though they could reduce both the incidence of potential resistant infections and the inappropriate use of antimicrobials (67). Another important but still under-investigated aspect is the patients/citizens' empowerment and engagement in the light of a shared decision-making, that is a key component of patient-centered health systems. An important point should be made also in regard to the evaluation of the technical value of vaccines and vaccination as the most of economic evaluations still adopt a narrow perspective on vaccines and vaccination benefits considering only health care cost savings and health gains. Nevertheless, a broader perspective should be undertaken, considering also outcome-related and care-related productivity gains, behavior-related output gains, health-based community externalities, risk reduction gains, reduction of comorbidities and other infections, and improvement in social equity (68). Nevertheless, also for this purpose, new tools are needed (68).

This systematic review has actually showed that there is room for improvements in regard to the concept of the value-based healthcare applied to vaccination.

To the best of our knowledge, this is the first systematic review that collated together the evidence on this topic shedding light on research gaps and needs. Evidence on the personal, allocative, technical, and societal value of vaccine(s)/vaccination published in the last decade and systematized in our review was described as foundation of a following consultation with international experts of the field. The result was the issuing of recommendations for research, decision-making, and public engagement to drive a value-based decision-making on vaccination (69). Further research activities would be desirable to address the same issues in low- and middle-income countries where the broad value of vaccines could be evaluated to a different extent.

Several limitations should be considered when interpreting results. Only articles published in English until May 27^th^, 2020 were included, which might have led to fail identifying all the available evidence on the topic up to now. Furthermore, a selection bias could not be completed ruled out even though the screening process was performed by five researchers independently and based on a lenient criterion (value mentioned in any part of the text). The search strings as well as the synthesis of evidence was informed by the content of the document published by the EXPH of the European Commission but this cannot exclude that some dimensions could have been missed. Furthermore, in order to keep the systematic review strictly focused on the concept of value, articles were not included if they did not explicitly mention that word. We are aware that a lot of primary studies addressing the different dimensions of value of vaccines and vaccination have been published up to now, but our goal was not to make a systematic review of studies focused on single dimensions. A quality assessment of the included articles was not performed; therefore, we could not assess the methodological correctness of the included studies. However, in our opinion, this does not impair our work as we wanted to provide an overview of pillars and dimensions assessed without addressing the robustness of methods used to do it. Eventually, the heterogeneity of evidence limits the possibility to further elaborate on information and data coming from the studies and issue definite findings. Nevertheless, in our opinion, this first depiction could help bringing forward the assessment and the appraisal of the value of vaccines and vaccination in the field of both academic research and supranational, national and local decision-making.

## Conclusions

Even though vaccines have contributed substantially to the reduction of the burden from infectious diseases, estimating their value is very complex. Each year, three million lives are saved thanks to vaccination. In developed countries, routine vaccinations have led to complete elimination or control of many diseases, drastically reducing their incidence, mortality rate and related complications (70). This article constitutes a comprehensive source of information on the value of vaccines/vaccinations and depicts the current evidence on the assessment of their personal, allocative, technical, and societal values. Increasing awareness of the true value of vaccines and vaccinations is of great importance as long as hesitation and underuse of vaccines may still lead to serious outbreaks. In fact, in a context of increasing pressure on healthcare budgets, vaccinations can contribute to the sustainability of health systems through a reduced and more efficient use of healthcare resources (71). As presented, the assessment of the whole value of vaccines and vaccinations needs to consider not just the direct impact on health and healthcare but also the wider impact on economic growth and societies. These wider impacts, although difficult to measure and still under-investigated, should be taken into consideration to better depict the whole values of vaccines and vaccinations and counteract vaccine hesitancy and misuse.

## Author Contributions

GEC, WR, and CdW conceptualized, designed, and supervised the study. AT, EC, MM, IG, and EB performed the data collection, screened the articles, and analyzed the data. AT and EC wrote the original draft of the manuscript that was revised and edited by GEC and CdW. All authors revised the manuscript and approved its submission for publication and certified that the work is original and their own.

## Funding

This study was funded by VIHTALI (Value In Health Technology and Academy for Leadership & Innovation), Spin-Off of Università Cattolica del Sacro Cuore, Rome, Italy. This paper reports the results of a VIHTALI project entitled “The value(s) of vaccination: building the scientific evidence according to a value-based healthcare approach”. The project was also funded by MSD.

## Conflict of Interest

GEC, EC, AT, IG, EB, MM, and CdW received a fee by VIHTALI (Value in Health Technology and Academy for Leadership & Innovation, Spin-Off of Università Cattolica del Sacro Cuore, Rome, Italy) for the scientific activities of the project entitled “The value (s) of vaccination: building the scientific evidence according to a Value-Based Healthcare approach.” WR was not paid for his time by him. The project was funded by MSD. The funders had no role in the design of the study, the collection, analysis and interpretation of data, or the writing of the manuscript.

## Publisher's Note

All claims expressed in this article are solely those of the authors and do not necessarily represent those of their affiliated organizations, or those of the publisher, the editors and the reviewers. Any product that may be evaluated in this article, or claim that may be made by its manufacturer, is not guaranteed or endorsed by the publisher.

## Footnotes

^1^ https://www.imf.org

^2^ https://www.who.int/gender-equity-rights/understanding/accessibility-definition/en/#:\sim:text=%E2%80%9Cis%20understood%20as%20the%20availability,services%20when%20they%20need%20them%E2%80%9D

^3^ https://www.who.int/health-topics/social-determinants-of-health#tab=tab_3
