# Supplementary material for: The Value(s) of Vaccination: Building the Scientific Evidence According to a Value-Based Healthcare Approach
Source: Front Public Health. 2022 Mar 9;10:786662. doi: 10.3389/fpubh.2022.786662 (PMC8963736; doi:10.3389/fpubh.2022.786662)
Supplement: Supplementary file 1 [file Data_Sheet_1.docx]

**Supplemental material**

The value(s) of vaccination: building the scientific evidence according to a value-based healthcare approach

**Appendix 1. Search strings**

| **Value Pillars** | **Database** | **Search strings** |
| --- | --- | --- |
|  |  |  |
| **Personal Value** | PUBMED | ((("patient reported outcome measures"[MeSH Terms] OR "patient reported outcome measures"[All Fields] OR "patient reported experience"[All Fields] OR preference[All Fields] OR participation[All Fields] OR Engagement[All Fields] OR ("empowerment"[MeSH Terms] OR "empowerment"[All Fields]) OR "commitment"[All Fields]) OR ("perception"[MeSH Terms] OR "perception"[All Fields]) OR ("attitude"[MeSH Terms] OR "attitude"[All Fields]) OR ("awareness"[MeSH Terms] OR "awareness"[All Fields]) OR efficacy[All Fields] OR effectiveness[All Fields] OR ("personal satisfaction"[MeSH Terms] OR "personal satisfaction"[All Fields] OR "satisfaction"[All Fields]) OR ("knowledge"[MeSH Terms] OR "knowledge"[All Fields]) OR "confidence"[All Fields]) OR complacency[All Fields] OR convenience[All Fields] OR "quality of life"[All Fields]) AND (("patients"[MeSH Terms] OR "patients"[All Fields] OR "patient"[All Fields]) OR citizen[All Fields]) AND (("immunisation"[All Fields] OR "vaccination"[MeSH Terms] OR "vaccination"[All Fields] OR "immunization"[All Fields] OR "immunization"[MeSH Terms]) OR ("vaccines"[MeSH Terms] OR "vaccines"[All Fields] OR "vaccine"[All Fields])) AND value[All Fields] |
|  | WoS | (((“Patient Reported Outcomes” OR "patient reported experience" OR preference OR participation OR Engagement OR Empowerment OR commitment OR perception OR attitude OR Awareness OR efficacy OR effectiveness OR satisfaction OR knowledge OR confidence OR complacency OR convenience OR “quality of life”) AND (patient OR citizen)) AND (immunization OR immunisation OR vaccination OR vaccine) AND value) |
| **Allocative Value** | PUBMED | (Access[All Fields] OR Accessibility[All Fields] OR Equity[All Fields] OR appropriateness[All Fields] OR appropriate[All Fields] OR allocation[All Fields] OR "innovation"[All Fields] OR "over use"[All Fields] OR "under use"[All Fields] OR "over utilization"[All Fields] OR "under utilization"[All Fields] OR program[All Fields] OR strategy[All Fields] OR strategies[All Fields] OR campaign[All Fields] OR "organisational model"[All Fields]) AND (("immunisation"[All Fields] OR "vaccination"[MeSH Terms] OR "vaccination"[All Fields] OR "immunization"[All Fields] OR "immunization"[MeSH Terms]) OR ("vaccines"[MeSH Terms] OR "vaccines"[All Fields] OR "vaccine"[All Fields])) AND value[All Fields] |
|  | WoS | ((Access OR Accessibility OR Equity OR appropriateness OR appropriate OR allocation OR innovation OR "over use" OR "under use" OR "over utilization" OR "under utilization" OR program OR strategy OR strategies OR campaign OR "organisational model") AND (immunization OR immunisation OR vaccination OR vaccine) AND value) |
| **Technical Value** | PUBMED | (("cost-benefit analysis"[MeSH Terms] OR "cost-benefit analysis"[All Fields]) OR "cost effectiveness"[All Fields] OR ("cost savings"[MeSH Terms] OR "cost savings"[All Fields] OR "cost saving"[All Fields]) OR ("costs and cost analysis"[MeSH Terms] OR "costs and cost analysis"[All Fields]) OR ("cost-benefit analysis"[MeSH Terms] OR "cost-benefit analysis"[All Fields] OR "cost benefit"[All Fields]) OR "cost utility"[All Fields]) OR "affordability"[All Fields] OR "hta"[All Fields] OR "fiscal impact"[All Fields] OR ("efficiency"[MeSH Terms] OR "efficiency"[All Fields]) OR "budget impact"[All Fields] OR ("models, economic"[MeSH Terms] OR "economic models"[All Fields] OR "economic model"[All Fields]) OR ("cost-benefit analysis"[MeSH Terms] OR "cost-benefit analysis"[All Fields]) OR "economic evaluation"[All Fields] OR "economic assessment"[All Fields] OR "economic appraisal"[All Fields] AND (("immunisation"[All Fields] OR "vaccination"[MeSH Terms] OR "vaccination"[All Fields] OR "immunization"[All Fields] OR "immunization"[MeSH Terms]) OR ("vaccines"[MeSH Terms] OR "vaccines"[All Fields] OR "vaccine"[All Fields])) AND value[All Fields] |
|  | WoS | ((“cost effectiveness” OR “cost saving” OR “cost efficient” OR “cost analysis” OR “cost benefit” OR “cost utility” OR affordable OR affordability OR HTA OR "fiscal impact" OR efficiency OR "budget impact" OR “economic model” OR “economic evaluation” OR "economic assessment" OR "economic appraisal") AND (immunization OR immunisation OR vaccination OR vaccine) AND value) |
|  | University of York CRD Database | ("cost effectiveness" OR "cost saving" OR "cost efficient" OR "cost analysis" OR "cost benefit" OR "cost utility" OR affordable OR affordability OR HTA OR "fiscal impact" OR efficiency OR "budget impact" OR "economic model" OR "economic evaluation" OR "economic assessment" OR "economic appraisal") AND (immunization OR immunisation OR vaccination OR vaccine) AND (value) |
| **Societal Value** | PUBMED | (("health impact assessment"[MeSH Terms] OR "health impact assessment"[All Fields]) OR "quality of life"[MeSH Terms] OR "quality of life"[All Fields] OR "population health"[MeSH Terms] OR "population health"[All Fields] OR "social impact"[All Fields]) OR "immunity, herd"[MeSH Terms] OR "herd immunity"[All Fields] OR "social responsibility"[MeSH Terms] OR "social responsibility"[All Fields] OR ("accountability"[All Fields] OR transparency[All Fields] OR appraisal[All Fields]) AND "decision making"[All Fields] OR ((wellbeing[All Fields] OR wealth[All Fields] OR protection[All Fields]) AND ("population"[MeSH Terms] OR "population"[All Fields] OR "population groups"[MeSH Terms] OR "population groups"[All Fields]) OR "community"[All Fields]) AND (("immunisation"[All Fields] OR "vaccination"[MeSH Terms] OR "vaccination"[All Fields] OR "immunization"[All Fields] OR "immunization"[MeSH Terms]) OR ("vaccines"[MeSH Terms] OR "vaccines"[All Fields] OR "vaccine"[All Fields])) AND value[All Fields] |
|  | WoS | (((“health impact assessment” OR “quality of life” OR “Population health” OR “social impact” OR “Herd Immunity” OR “Social responsibility” OR (accountability OR transparency OR appraisal) AND “decision making”) OR ((wellbeing OR wealth OR protection) AND (population OR community))) AND (immunization OR immunisation OR vaccination OR vaccine) AND value) |

**Appendix 2. PRISMA flowcharts**

**Figure 1. Personal pillar**

**Figure 2. Allocative pillar**

**Figure 3. Technical pillar**

**Figure 4. Societal pillar**
